# Large Language Models can Identify the Presence of MASH and Extract VCTE Measurements from Unstructured Documentation

**DOI:** 10.1007/s10620-025-09539-1

**Published:** 2025-11-08

**Authors:** Aryana T. Far, Aryan Ayati, Jordan Guillot, Shadera Azzam, Vivek A. Rudrapatna, Jin Ge

**Affiliations:** 1 Division of Gastroenterology and Hepatology, Department of Medicine, University of California – San Francisco, San Francisco, CA USA; 2 Bakar Computational Health Sciences Institute, University of California – San Francisco, San Francisco, CA USA

**Keywords:** Fibrosis assessment, Natural language processing, Clinical informatics, Risk stratification, Unstructured clinical data, Digital phenotyping

## Abstract

**Introduction:**

Metabolic dysfunction-associated steatohepatitis (MASH) is a leading cause of cirrhosis. Vibration Controlled Transient Elastography (VCTE) measurements are often captured in text-based reports and not readily accessible for clinical research. Large language models (LLMs) show promise for curating information from unstructured documentation, but their efficiency for MASH and VCTE extraction are unclear.

**Methods:**

We used a cohort of 493 patients with compensated MASH cirrhosis. We compared the abilities of GPT-4o and Claude 3.5 Sonnet for identifying the presence of MASH and extracting maximum VCTE stiffness and Controlled Attenuation Parameter (CAP) measurements from clinical documentation. We ran a cost analysis of the LLMs. As exploratory analysis, we used LASSO-Cox to associate LLM-extracted features with death or decompensation.

**Results:**

For identifying MASH in clinical notes, GPT-4o and Claude 3.5 achieved F1-scores of 90.5% and 80.0%. For identifying peak VCTE measurements, GPT-4o achieved 99.3% and 99.1% accuracies for stiffness and CAP, while Claude 3.5 achieved 93.3% and 94.1% accuracies. LLM extraction of one variable required ~ 2000 tokens per note, with a cost of ~ $0.012/note for GPT-4o and ~ $0.014/note for Claude 3.5. In LASSO-Cox regressions, VCTE stiffness (HR 1.03, 95% CI 1.01–1.05, *p* = 0.016) and CAP score (HR 0.99, 95% CI 0.99–1.00, *p* = 0.029) were statistically significant predictive variables for death or decompensation.

**Conclusions:**

LLMs can extract MASH presence and VCTE parameters from documentation with high accuracy and low cost. When incorporated into survival analyses, LLM-extracted variables are associated with important clinical outcomes. Given the growing availability of LLMs, liver diseases researchers should incorporate these methods to facilitate real-world studies.

**Supplementary Information:**

The online version contains supplementary material available at 10.1007/s10620-025-09539-1.

## Introduction

Metabolic dysfunction-associated steatohepatitis (MASH) is a leading cause of liver-related morbidity and mortality worldwide [1, 2]. Accurate identification of MASH is essential for both clinical decision-making and research; however, it remains challenging due to under-coding and inconsistent documentation in electronic health records (EHRs) [3]. Moreover, key diagnostic data for risk stratification in MASH, like Vibration Controlled Transient Elastography (VCTE) values, are semi-structured, often captured as free text rather than as structured data, limiting their use in large cohort studies [4, 5]. Bridging this gap by converting unstructured data into analyzable structured formats is therefore an unmet need, and addressing it could significantly improve population-level management of MASH.

Natural language processing (NLP) and emerging advanced techniques using large language models (LLMs) enable the automated extraction of structured data from unstructured clinical notes. Our group previously developed a rule-based NLP algorithm, “NASHDetection,” which identifies MASH from clinical notes with up to 86% accuracy [6]. We have also pioneered the use of LLM-based methods to identify diagnoses of cirrhosis and its complications from hospitalization discharge summaries, with performance exceeding traditional code-based identification methodologies [7]. These results demonstrate that LLM-based NLP, which reaches the accuracy of human reviewers in many cases, could be expanded to other data extraction tasks in liver disease research.

In this study, we evaluated the performance of LLMs in identifying both (1) the presence of MASH in hepatology and liver transplant documentation and (2) peak VCTE measurement values in patients. For the identification of MASH, we compared the performance of the LLMs with our existing rule-based “NASHDetection” algorithm. As an exploratory analysis of the value of LLM-extracted variables in clinical research, we ran regression models combining LLM-extracted VCTE measurements with readily available structured variables from EHRs.

## Methods

### Study Cohort and Data Sources

We used a cohort of adult patients with compensated MASH cirrhosis previously established in a published study. That work identified 493 patients seen at the University of California, San Francisco (UCSF) Medical Center between January 1, 2012, and January 1, 2024. Both unstructured hepatology and transplant clinic notes as well as structured clinical variables were extracted from the UCSF De-Identified Clinical Data Warehouse. In the original study, ground truth for the presence of MASH in a note was established through the manual review of 150 clinical notes [6]. For the present study, we utilized this cohort for further analyses.

### MASH Detection and Evaluation

We used protected health information (PHI)-compliant instances of LLMs to identify the presence of MASH in individual clinical notes. We specifically used OpenAI’s GPT-4o and Anthropic’s Claude 3.5 Sonnet, deployed within UCSF’s private and secure artificial intelligence (AI) Versa platform. Model prompting and parameterization followed frameworks established in our prior work on large-scale LLM extraction of cirrhosis presence from UCSF’s de-identified clinical notes [7]. We iteratively refined the prompt to explicitly address edge cases, including recent nomenclature changes (NAFLD, NASH, MASLD, MASH) [8], negated mentions (e.g., “no evidence of MASH”), suspected or presumed diagnoses, multi-etiology liver disease, post-transplant occurrences, and cases where MASH could be inferred from treatment (e.g., vitamin E, resmetirom, or GLP-1 agonists). Both models were run in a zero-shot setting with temperature 0.7 and otherwise default parameters. The temperature of 0.7 has proven to be optimal in similar prior work involving deterministic extraction of fixed diagnostic labels [7], and commonly serves as the default setting in commercially implemented LLMs like ChatGPT. No fine-tuning was performed. Prompts are provided in the Supplemental Materials.

To evaluate GPT-4o and Claude 3.5 in identifying MASH, we used the prior manual review of 150 notes as the gold standard. Two reviewers (a physician and a clinically trained non-physician reviews) labeled each note, and cases were adjudicated and confirmed by a hepatology sub-specialist. We compared the LLMs’ performances to the rule-based “NASHDetection” algorithm. We assessed the performance of all three methods using positive predictive value (PPV), sensitivity, specificity, accuracy, F1-score, and negative predictive value (NPV). We calculated confidence intervals for each performance metric via normal approximation. We also conducted qualitative error analysis on cases where there was discordance between ground truth and LLM outputs.

### VCTE Value Extraction and Evaluation

We used GPT-4o and Claude 3.5 to identify peak VCTE values in patients. At UCSF, VCTE is performed using the Echosens FibroScan device, which reports both liver stiffness and CAP; we specifically queried for these measurements in our cohort [9]. These results were documented in UCSF’s unstructured clinical notes, and because of inconsistencies and de-identification, regular expressions alone were insufficient for extraction. To identify candidate notes, we filtered for notes containing key VCTE terms and units. We then prompted GPT-4o and Claude 3.5 to extract liver stiffness and controlled attenuation parameter (CAP) values, using the same methodology as our MASH presence extraction. Prompts are provided in the Supplemental Materials. For patients with multiple extracted VCTE values, we retained the maximum, consistent with prior literature emphasizing the clinical relevance of peak measurements [10–12].

To evaluate the LLMs’ extraction of peak VCTE values for patients, we conducted manual chart review on 100% of patients identified as having available results. We calculated accuracy with confidence intervals and root mean square error (RMSE).

### Cost Analysis of Data Extraction

To estimate the cost involved in data extraction for the whole cohort, we approximated LLM efficiency based on tokens, model pricing, and time estimates. We compared the cost of LLMs with that of rule-based methods and manual review.

### Exploratory Survival Analysis

As an exploratory analysis, we examined whether extracted VCTE values were prognostic of clinical outcomes. Analyses were limited to the subset of patients with available VCTE results. We performed Cox proportional hazards modeling with L1 regularization, using a composite endpoint of decompensation (ascites, spontaneous bacterial peritonitis, variceal hemorrhage, or hepatic encephalopathy) or all-cause mortality. We incorporated VCTE stiffness VCTE CAP values as continuous predictors alongside structured clinical covariates, including demographics, laboratory values, comorbidities, and medications. For each time-to-event model corresponding to the measurement covariates, start dates were considered as the date of measurement. Given the limited sample size of patients with available VCTE measurements, these analyses were intended as exploratory rather than definitive.

## Results

### Cohort Characteristics

The cohort included 493 patients (61.5% female) with a median age of 58 years (interquartile range [IQR] 49–66), median BMI of 33.5 kg/m^2^ (IQR 29.4–36.5), and median MELD score of 8.0 (IQR 6.4–9.9). The most common comorbid conditions include hypertension (65%), diabetes (54%), and cardiovascular disease (16%). Please see Table 1 for full demographic, laboratory, and clinical characteristics.

**Table 1:** Baseline characteristics of full cohort

Variable	Full cohort
Gender = Female, N (%)	303 (61.5)
Age, Median (IQR)	58 (49, 66)
Race	
Non-Hispanic White, N (%)	187 (37.9)
Hispanic or Latino, N (%)	168 (34.1)
Asian, N (%)	65 (13.2)
Black, N (%)	8 (1.6)
Native or Other, N (%)	65 (13.2)
Body Mass Index (BMI), Median (IQR)	33.5 (29.4, 36.5)
Labs	
Albumin, Median (IQR)	4.0 (3.8, 4.2)
Total Bilirubin, Median (IQR)	0.9 (0.6, 1.2)
INR, Median (IQR)	1.1 (1.0, 1.2)
Serum Sodium, Median (IQR)	138.2 (137.1, 140.0)
Creatinine, Median (IQR)	0.8 (0.7, 1.0)
MELD Score, Median (IQR)	8.0 (6.4, 9.9)
FIB-4, Median (IQR)	2.1 (1.5, 2.8)
GPT-derived Fibroscan Stiffness	10.5 (7.1, 16.3)
GPT-derived CAP Score	324.0 (282.5, 358.0)
Comorbidities	
Diabetes, N (%)	268 (54.4)
Hypertension, N (%)	320 (64.9)
Sleep Apnea, N (%)	87 (17.6)
Cardiovascular Disease, N (%)	81 (16.4)
Chronic Kidney Disease, N (%)	25 (5.1)

### MASH Detection Performance

We evaluated the performances of GPT-4o, Claude 3.5, and the rule-based “NASHDetection” algorithm against the subset of N = 150 notes that underwent manual chart review. As shown in Table 2, GPT-4o demonstrated the most balanced performance, achieving an F1-score of 90.5% and an accuracy of 92.0%, with high PPV (91.9%) and sensitivity (89.1%). Claude 3.5 showed the highest PPV (95.7%) and specificity (97.8%), but its lower sensitivity (68.8%) led to a reduced F1-score of 80.0%, suggesting it was more conservative and less likely to identify true positive cases. In contrast, the rule-based “NASHDetection” algorithm showed strong sensitivity (89.1%) but lower PPV (80.3%), resulting in an F1-score of 84.4%. See Table 2 for all performance metrics with confidence intervals.

**Table 2:** Evaluation of GPT-4o, Claude 3.5, and “NASHDetection” algorithm Versus Gold-Standard Manual Chart Review (N = 150 notes) in detecting MASH

NLP method	PPV [95% CI]	Sensitivity [95% CI]	F1 Score [95% CI]	Accuracy [95% CI]	NPV [95% CI]	Specificity [95% CI]
GPT-4o	91.94 [87.63–95.25]	89.06 [83.99–94.13]	90.48 [85.71–95.25]	92.00 [87.82–96.18]	92.05 [87.88–96.22]	94.19 [90.88–97.50]
Claude 3.5	95.65 [92.23–99.07]	68.75 [61.24–76.26]	80.00 [73.39–86.61]	85.33 [80.00–90.66]	80.77 [74.20–87.34]	97.67 [95.15–100.00]
NASHDetection	80.28 [73.91–86.65]	89.06 [83.99–94.13]	84.44 [78.47–90.41]	86.00 [80.21–91.79]	91.13 [86.08–96.18]	83.72 [77.29–90.15]

We conducted qualitative error analyses of notes misclassified by either LLMs, defined as error by GPT-4o or Claude 3.5 (N = 24). In 16 of these cases (67%), the models disagreed with each other, while in 8 notes both GPT-4o and Claude 3.5 made the same misclassification. Most errors occurred in cases describing hepatic steatosis or metabolic-associated fatty liver disease (MASLD) without explicit mention of MASH, or where diagnostic uncertainty and negation (e.g., ‘possible cirrhosis,’ ‘recommend biopsy to confirm’) obscured the true diagnosis. 19 (79%) of these notes also involved mixed or unclear etiologies under evaluation (e.g., autoimmune, viral, or metabolic).

### VCTE Value Extraction Performance

GPT-4o identified 149 patients with at least one stiffness value and 115 with at least one CAP value, while Claude 3.5 identified 149 patients with at least one stiffness value and 118 with at least one CAP value. After consolidating to the maximum VCTE value per patient, median GPT-derived peak values were 10.5 kPa (IQR: 7.1–16.3) for stiffness and 324 dB/m (IQR: 282.5–358) for CAP, while Claude-derived medians were 10.5 kPa (IQR: 7.2–16.3) and 325 dB/m (IQR: 286.0–363.5), respectively.

We evaluated the performances of GPT-4o and Claude 3.5 against 100% manual review of patients with available VCTE values. GPT-4o achieved 99.3% accuracy for stiffness (N = 149 patients) and 99.1% for CAP (N = 115), whereas Claude 3.5 achieved 93.3% (N = 149) accuracy for stiffness and 94.1% for CAP (N = 118). Among patients with ground truth values, the root mean squared error (RMSE) was 0.3 kPa for GPT-derived stiffness and 3.5 dB/m for GPT-derived CAP, compared with 0.4 kPa for Claude-derived stiffness and 6.5 dB/m for Claude-derived CAP. Summary statistics and evaluation metrics for all extracted VCTE values are shown in Table 3.

**Table 3:** Summary statistics and evaluation of LLM-derived VCTE measurements

LLM-extracted VCTE measurement	Number of patients with available values	Accuracy [95% CI]	RMSE	Median (IQR)
GPT-4o Stiffness	149	99.3 [97.9–100]	0.3 kPa	10.5 kPa (7.1–16.3 kPa)
GPT-4o CAP	115	99.1 [97.4–100]	3.5 dB/m	324 dB/m (282.5–358 dB/m)
Claude 3.5 Stiffness	149	93.3 [89.3–97.3]	0.4 kPa	10.5 kPa (7.2–16.3 kPa)
Claude 3.5 CAP	118	94.1 [89.8–98.3]	6.5 dB/m	325 dB/m (28–363.5 dB/m)

### Cost Analysis of Data Extraction

Scaled to the whole cohort, applying the three LLM prompts to relevant notes for extracting MASH presence and VCTE measurements would require a total of ~ 6.5 million tokens. This corresponds to an average of ~ 2000 tokens per note. Thus, extracting the three variables for the whole cohort would cost approximately $60 for GPT-4o and $70 for Claude 3.5. Extracting a single variable from an individual note would cost an average of about $0.012 with GPT-4o and $0.014 with Claude 3.5.

In terms of time efficiency, GPT-4o was about 20% faster than Claude 3.5. Compared with manual chart review, which had an average rate of 20 notes/hour, LLM extraction exceeded 1,000 notes/hour. Furthermore, compared with rule-based NLP algorithms such as “NASHDetection,” which require extensive development and tuning [13], the LLMs’ prompt development and tuning time was minimal.

### Exploratory Analysis of Survival

VCTE stiffness and CAP variables used in outcome analyses were derived from GPT-4o, as it demonstrated the best performance in identifying MASH and extracting measurement values. In the overall cohort of patients with compensated MASH cirrhosis, there were 140 events of death or decompensation. Among the 149 patients with GPT-derived stiffness values, 20 events occurred, while among the 115 patients with CAP values available, 17 events occurred. In the LASSO-Cox models for the composite outcome of death or decompensation, both LLM-derived liver stiffness and CAP were maintained as predictive variables and reached statistical significance at the 5% level. Liver stiffness was associated with a hazard ratio of 1.03 per 1-unit increase in kPa [95% CI: 1.01–1.05, *p* = 0.016], and CAP was associated with a hazard ratio of 0.99 per 1-unit increase in dB/m [95% CI: 0.99–1.00, *p* = 0.029]. These findings should be interpreted with caution given the relatively small number of events in each subgroup. Hazard ratios for all variables retained in these models are reported in Tables 4 and 5.

**Table 4:** Hazard ratios for selected variables in the VCTE stiffness LASSO-Cox model for the composite outcome (N = 149 patients)

Covariate	HR [95% CI]	*p* value
Demographics: Age	0.95 [0.65–1.38]	0.786
Socioeconomic: ADI Mean State	0.92 [0.65–1.31]	0.639
Lab: MELD Score	1.30 [1.01–1.66]	0.038
Lab: FIB-4 Score	1.37 [1.00–1.88]	0.052
Comorbidity: GERD	1.30 [0.64–2.66]	0.472
Comorbidity: Hyperlipidemia	0.55 [0.25–1.19]	0.127
Comorbidity: Sleep Apnea	1.87 [0.88–4.01]	0.105
Medication: Calcium Channel Blocker	4.47 [1.64–12.22]	0.004
Medication: Vitamin E	1.72 [0.69–4.27]	0.244
Fibroscan stiffness	1.03 [1.01–1.05]	0.016
Demographics: Baseline Year > = 2020	0.73 [0.36–1.51]	0.399

**Table 5:** Hazard ratios for selected variables in the VCTE CAP score LASSO-Cox model for the composite outcome (N = 115 patients)

Covariate	HR [95% CI]	*p* value
Demographics: Age	0.88 [0.57–1.36]	0.564
Demographics: Driving Distance (km)	1.28 [0.83–1.97]	0.259
Socioeconomic: ADI Mean State	0.96 [0.61–1.48]	0.838
Lab: MELD Score	1.36 [1.04–1.78]	0.027
Lab: FIB-4 Score	1.46 [0.99–2.15]	0.056
Comorbidity: GERD	1.55 [0.69–3.48]	0.287
Comorbidity: Hypertension	1.78 [0.63–5.03]	0.280
Comorbidity: Sleep Apnea	2.08 [0.85–5.07]	0.108
Medication: Calcium Channel Blocker	4.19 [1.23–14.27]	0.022
Medication: Sulfonylurea	2.08 [0.61–7.07]	0.239
Fibroscan CAP score	0.99 [0.99–1.00]	0.029
Demographics: Baseline Year > = 2020	0.87 [0.39–1.96]	0.738
Demographics: Overweight	0.55 [0.20–1.46]	0.230

## Discussion

This study demonstrates that LLMs can accurately identify both the presence of MASH in clinical notes and peak VCTE values in patients. For detection of MASH, both GPT-4o and Claude 3.5 outperformed traditional NLP methods and approached the accuracy of expert review. Furthermore, the LLMs offered greater efficiency than manual review and lower development burden compared with rule-based NLP. As an exploratory analysis, we incorporated LLM-extracted VCTE values into survival models and found that liver stiffness and CAP scores were independently associated with a composite endpoint of decompensation and mortality. Although the prognostic value of VCTE measurements is well established, our findings suggest that LLMs can effectively extend such insights to large, retrospective real-world datasets. This approach enables population-level studies of disease progression and risk stratification by converting unstructured clinical documentation into analyzable variables.

In our validation evaluations, we observed performance differences between the "NASHDetection” algorithm, GPT-4o, and Claude 3.5 in identifying the presence of MASH. “NASHDetection” was highly sensitive but prone to over-inclusion. Claude 3.5, in contrast, was more conservative and often missed true cases. GPT-4o offered a balance of sensitivity and specificity. In extracting VCTE measurements, Claude 3.5 was more prone to error than GPT-4o. These differences likely reflect each model’s core strengths: in computer science literature, Claude 3.5 has been observed to excel at structured tasks like programming and code generation, while GPT-4o and OpenAI’s o1 models have been shown to have stronger performance with reasoning and nuanced language [14, 15]. This underscores the importance of selecting models aligned with task demands, particularly in clinical NLP applications.

Several limitations should be acknowledged. The retrospective design introduces potential biases. LLM extraction results may be affected by variability in clinical documentation and the de-identification process. Results of the survival analysis on patients with VCTE measurements should be considered with caution. Particularly, in this exploratory analysis, lower CAP values were associated with worse outcomes. This association may reflect “burn-out” steatohepatitis – defined as patients with advanced fibrosis and low hepatic fat accumulation – which is associated with adverse outcomes [16]. However, the limited sample size of patients with available VCTE measurements reduces generalizability. Selection bias is also likely, as VCTE procedures are more commonly performed in individuals with suspected advanced disease.

Future work should expand this approach to larger and more diverse populations and explore LLM-based extraction of additional liver-related diagnostic and prognostic variables. This would further enable scalable, data-driven insights across the full spectrum of chronic liver disease.

## Supplementary Information

Below is the link to the electronic supplementary material.

Supplementary file1 (DOCX 20 KB)

## Data Availability

No datasets were generated or analysed during the current study.

## Abbreviations

CAP: Controlled attenuation parameter
LLM: Large language model
MASH: Metabolic-associated steatohepatitis
NLP: Natural language processing
NPV: Negative predictive value
PHI: Protected health information
PPV: Positive predictive value
RMSE: Root mean squared error
UCSF: University of California, San Francisco
VCTE: Vibration controlled transient elastography
