# Supplementary material for: Large Language Models can Identify the Presence of MASH and Extract VCTE Measurements from Unstructured Documentation
Source: Dig Dis Sci. 2025 Nov 8;71(4):1505–11. doi: 10.1007/s10620-025-09539-1 (PMC13144198; doi:10.1007/s10620-025-09539-1)
Supplement: Supplementary file 1 — Supplementary file1 (DOCX 20 KB) [file 10620_2025_9539_MOESM1_ESM.docx]

**Large language models can identify the presence of MASH and extract VCTE measurements from unstructured documentation**

Aryana T. Far, Aryan Ayati, Jordan Guillot, Shadera Azzam, Vivek A. Rudrapatna, Jin Ge

**Table of Contents**

**Supplementary Methods** ........................................................................................................ p. 2

**Prompt 1. LLM Prompt for MASH Presence.** ..................................................................... p. 3

**Prompt 2. LLM Prompt for VCTE Stiffness Measurement.** .............................................. p. 3

**Prompt 3. LLM Prompt for VCTE CAP Score.** .................................................................. p. 3

**Supplementary Methods**

All methods used for identifying outcomes and processing data are described in the main Methods section. Additional analyses shown here (e.g., LLM prompts) used the same definitions and procedures unless otherwise specified.

**Prompt 1. LLM Prompt for MASH Presence.**

“Carefully examine the progress note for a diagnosis of non-alcoholic steatohepatitis (NASH) or metabolic dysfunction-associated steatohepatitis (MASH). This can include explicit references to 'NASH' or 'MASH' or related descriptions such as 'fatty liver with inflammation,' or 'steatohepatitis' not associated with alcohol use. The mention of these phrases should not be in the context of negation, such as 'no evidence of MASH.’ The mention of NASH/MASH diagnosis could be a ‘presumed’ or ‘suspected’ diagnosis. If the note mentions non-alcoholic fatty liver disease (NAFLD) or metabolic dysfunction-associated steatotic liver disease (MASLD) and it is accompanied with a treatment for NASH/MASH such as vitamin E, resmetirom, or GLP-1 agonist, then this is equivalent to a diagnosis. If the note mentions multiple etiologies of liver disease, including NASH/MASH, then this would count as ‘True.’ If the note mentions the patient is being evaluated for potential NASH/MASH with a list of potential differential diagnoses or consideration of assessment for fibrosis with liver biopsy or FibroScan, then this would count as ‘False.’ If the occurrence of NASH/MASH is after (or status post) a liver transplant, then this would count as ‘False.’ Return ‘True’ if NASH/MASH is one of the diagnoses in the document, otherwise return ‘False.’”

**Prompt 2. LLM Prompt for VCTE Stiffness.**

“From the following clinical note, extract the largest FibroScan stiffness value reported (e.g., in kPa), and the date it was measured, if available.

Return your answer in the following JSON format: {‘fibroscan_stiffness’: number or null, ‘fibroscan_date’: ‘YYYY-MM-DD' or null}
 Requirements:

- Include decimals.
- Do not include units.
- Return the largest numeric stiffness value if multiple are present.
- Use only values that clearly refer to a FibroScan stiffness or liver stiffness measurement.
- If no valid value or date is available, return null.

**Prompt 3. LLM Prompt for VCTE CAP:**

“From the following clinical note, extract the most severe CAP score (Controlled Attenuation Parameter) reported, and the date it was measured, if available.

Return your answer in the following JSON format: {‘cap_score’: int or null, ‘cap_date’: ‘YYYY-MM-DD' or null}
 Requirements:

- Do not include units.
- Return the highest CAP score value if multiple are present.
- Use only values clearly identified as CAP score or Controlled Attenuation Parameter.
- If no valid value or date is available, return null.”
